# Rationally assembled albumin/indocyanine green nanocomplex for enhanced tumor imaging to guide photothermal therapy

**DOI:** 10.1186/s12951-020-00603-8

**Published:** 2020-03-17

**Authors:** Feifei An, Zhao Yang, Meichen Zheng, Ting Mei, Guowei Deng, Ping Guo, Yanan Li, Ruilong Sheng

**Affiliations:** 1 Institute of Medical Engineering, Department of Biophysics, School of Basic Medical Science, Health Science Center, Xi’an Jiaotong University, No.76 Yanta West Road, Xi’an, 710061 Shaanxi People’s Republic of China; 2 College of Chemistry and Life Science, Institute of Functional Molecules, Chengdu Normal University, Chengdu, 611130 China; 3 College of Medical Imaging, Shanxi Medical University, Taiyuan, 030001 Shanxi People’s Republic of China; 4 Department of Radiology, First Hospital of Shanxi Medical University, Taiyuan, 030001 Shanxi People’s Republic of China; 5 CQM-Centro de Quimica da Madeira, Universidade da Madeira, Campus da Penteada, 9000-390 Funchal, Madeira Portugal

**Keywords:** Indocyanine green, Albumin, Near-infrared, Photothermal therapy, Theranostics

## Abstract

Herein, a novel phototheranostic nanocomplex that is self-assembled from bovine serum albumin (BSA) and indocyanine green (ICG) is developed for enhanced near-infrared (NIR) fluorescence imaging, which benefits the guidance on in vivo cancer photothermal therapy (PTT). The study confirms that the binding of ICG with the bind sits on the albumin will result in improved hydrolytic stability and high photoluminescence quantum yield (PLQY). The ICG loading ratio in the nanocomplex is optimized and confirms the loading ratio of 0.5% ICG to be the optimal content. The optimized ICG–BSA nanocomplex (ICG–BSA NC) possesses a higher PLQY of 16.8% than that of free ICG (2.7%). The high PLQY and efficient passive targeting ability of ICG–BSA NC help improve its in vivo tumor accumulation and NIR fluorescence imaging significantly. Under laser irradiation, efficient PTT with obvious tumor growth suppression on a triple negative breast tumor model can be observed in the ICG–BSA NC treated group.

## Background

Cancer therapy assisted with a theranostic agent has attracted intensive interests in recent years for improved therapeutic effect [1–3]. An ideal theranostic agent is expected to possess several characteristics, including biocompatible compositions, imaging ability, therapy ability, tumor targeting ability [4]. Indocyanine green (ICG) is undoubtedly a superior candidate for cancer theranostics due to its proved safety in clinic, near-infrared (NIR) fluorescence imaging (FI) ability and photothermal therapy (PTT) ability [5]. As a small molecule, ICG itself fails to have enough accumulation at the tumor for effective NIR imaging guided PTT. In order to improve the tumor accumulation, ICG is generally loaded in a nanoparticle to utilize the well-known passive & active targeting abilities of nanoparticles [6–9]. The incorporation of ICG into a nanoparticle has demonstrated effective delivery of ICG to the tumor for in vivo cancer theranostics [10, 11].

The importance of using a biocompatible carrier material has been well-recognized for potential clinical translation of nanomedicine [12, 13]. As an endogenous circulating proteins in blood and a transporter for various exogenous compounds within the vasculature, albumin shows relatively long circulation half-life and favorable biocompatibility [14]. In recent years, albumin has been intensively explored to construct nanocarrier for delivering drug and contrast agent to the tumor [15, 16]. There are two binding sites on the albumin, which could be used to load small molecules [17]. It is discovered that rational incorporation of a dye into the albumin-based nanoparticle could help improve the photoluminescence quantum yield (PLQY) of the dye, which benefits the precise tumor imaging and therapy with external stimulus [18, 19]. The combination of albumin and ICG in a nanoparticle for NIR fluorescence imaging guided PTT with external laser irradiation has been also reported [20, 21], however, the attempt to optimize the PLQY of ICG to improve the tumor diagnosis precision has not been explored. Therefore, it is meaningful to assemble ICG and albumin in a rational way to construct an optimized nanoplatform for augmenting the effects of tumor diagnosis and image-guided cancer therapy.

**Fig. 1:**
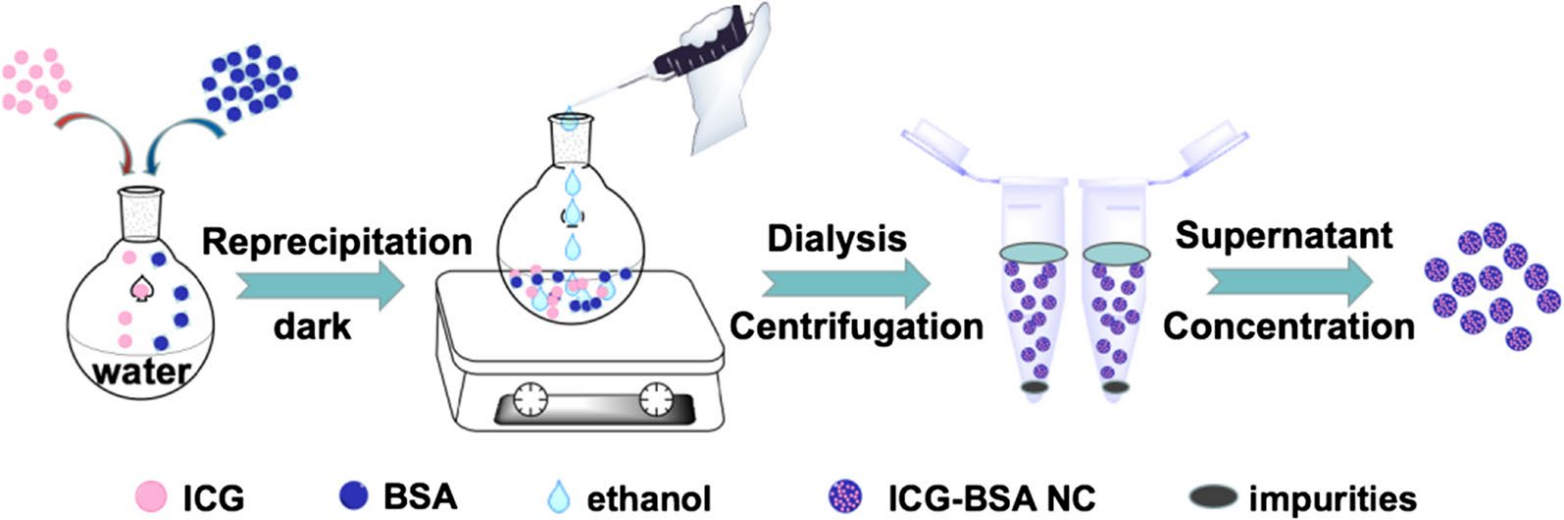
The scheme of preparing ICG–BSA NC

In this work, we report an optimized phototheranostic nanocomplex (NC) that is rationally assembled from bovine serum albumin (BSA) and ICG for enhanced NIR fluorescence imaging guided PTT on tumor. The NC is prepared by the self-assembling of BSA and ICG via nanoprecipitation method (Fig. 1). The prepared NC is optimized by measuring and comparing the PLQYs of a series of NC that having different ICG loading ratio. The results shows that a loading ratio of 0.5% ICG is the optimal content with highest PLQY and improved hydrolytic stability. The prepared 0.5% ICG–BSA NC possesses a much higher PLQY (16.8%) than that of free ICG (2.7%). In vitro study demonstrates efficient cell imaging capacity and PTT effect. Further in vivo study shows significant tumor accumulation and efficient NIR fluorescence imaging with high PLQY and passive targeting ability of ICG–BSA NC. Under laser irradiation, efficient PTT with obvious tumor growth suppression on a triple negative breast tumor model can be observed in ICG–BSA NC treated group.

## Results and discussion

### Synthesis and characterization of the ICG–BSA NC

**Fig. 2:**
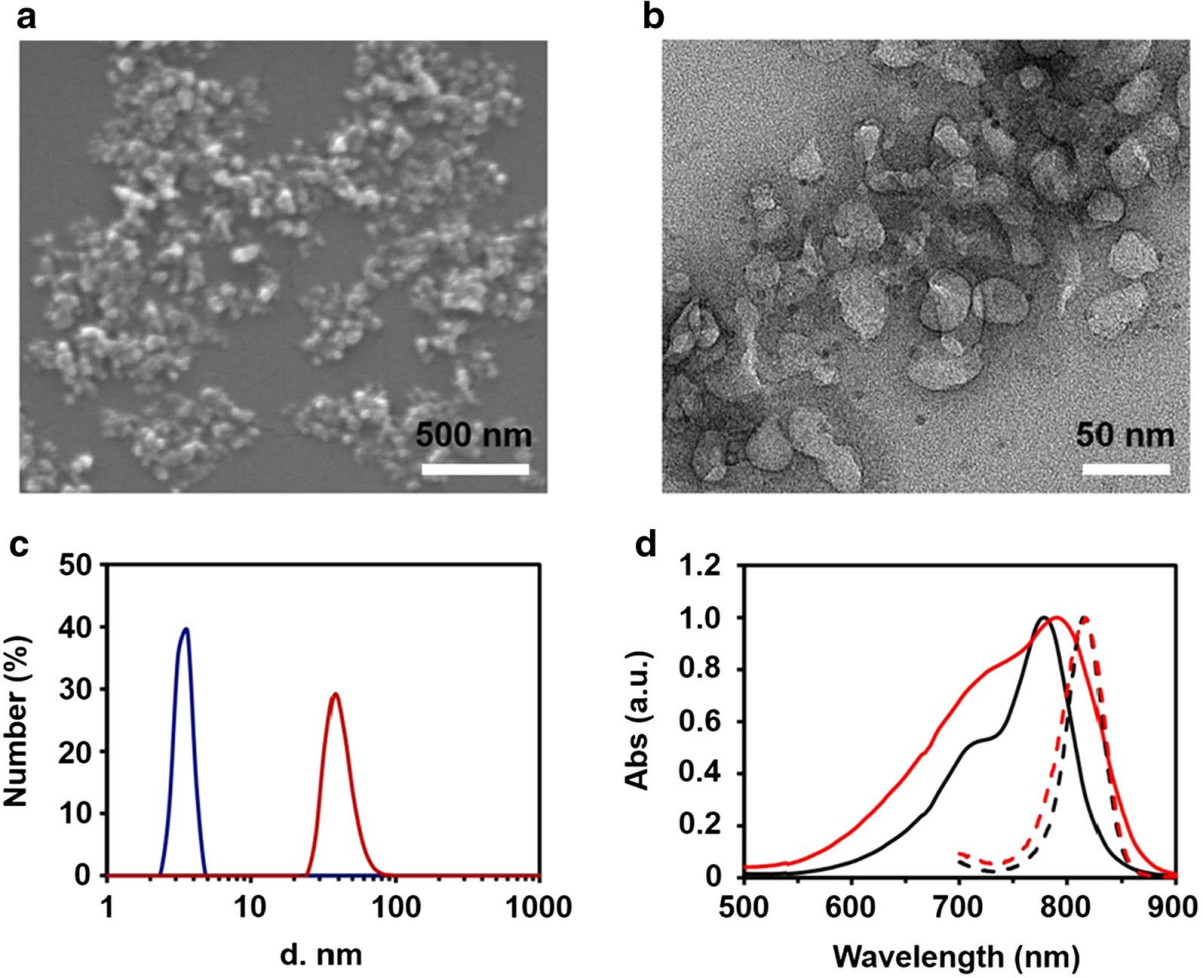
**a** The SEM characterization of the prepared ICG–BSA NC. **b** The TEM characterization of the prepared ICG–BSA NC. **c** The DLS characterization of the prepared ICG–BSA NC (red) and single BSA (blue). **d** The UV–Vis–NIR absorption spectra and fluorescence emission spectra of free ICG molecule (black line) and ICG–BSA NC (red line) in 1× PBS (pH 7.4)

The fabrication procedure of ICG–BSA NC was schematically described in Fig. 1. Free ICG and BSA are dispersed evenly in water to form a mixed solution. The introduction of ethanol reduces the albumin solubility and results in the formation of ICG–BSA complex by means of hydrogen bond and hydrophobic interactions. The bulk precipitate is removed by centrifuge. As shown in the SEM image (Fig. 2a), the obtained ICG–BSA NC exhibits uniformly spherical structure with an average diameter of ~ 35 nm, which is further confirmed by the TEM characterization (Fig. 2b). The DLS analysis is also used and shows that the ICG–BSA NC has a size distribution of ~ 41 nm. The size is slightly larger than those obtained from SEM and TEM due to the hydration effect during the DLS characterization. In addition, the nanocomplex dried and shrunk during the sample preparation for SEM and TEM characterizations, which resulted in a smaller size. In contrast, the single albumin protein exhibits a size distribution of ~ 3.4 nm (Fig. 2c). The size of obtained ICG–BSA NC is suitable for in vivo tumor targeting via enhanced permeation and retention (EPR) effect [22, 23]. The aqueous solution of ICG molecule exhibits strong absorption peak at 780 nm in the UV–vis–NIR spectra (Fig. 2d). In contrast, the aqueous solution of ICG–BSA NC exhibits an absorption peak at 791 nm, which is slightly red-shifted compared with that of ICG molecule in water. The red-shift of the absorption peak indicate an interaction between ICG molecule and the BSA molecule, which is also well-observed in some other system between dye and BSA [24]. The interaction between ICG molecule and BSA molecule indicates the successful introduction of ICG into the BSA-based nanocomplex. Both absorption and emission peaks of ICG–BSA NC locate at the NIR wavelength region (700–900 nm), which is called biological window for light penetration to deep tissue and good optical imaging. Therefore, the optical properties of the prepared ICG–BSA NC are very suitable for in vivo NIR tumor imaging guided photothermal therapy with 808 nm laser.

### Optimization of ICG loading and fluorescence performance

A series of ICG–BSA NCs with increasing ICG content were prepared for screening the optimal fluorescence property. As shown in Table 1, the ICG content in the ICG–BSA NCs increases in the range of 0.1% to 5% (wt), while the PLQY does not increase linearly. The PLQY is positively correlated with ICG content when less than 0.5%, which reaches a maximal PLQY of 0.168 at the content of 0.5%. Then, the PLQY gradually decreases along with the increased ICG content, the PLQYs of 1%, 2% and 5% ICG contents are 0.157, 0.143 and 0.113, respectively. The result reveals that ICG–BSA NC with an ICG content of 0.5% could be the optimal composition for NIR FI guided photothermal therapy. It is known that there are binding sites on the albumin [17]. According to the calculation, the molar ratio (ICG:BSA) of the group with a loading ratio of 0.5% (wt) is < 1.0, in which ICG could avoid aggregation induced fluorescence quenching at the binding site.

**Table 1:** The PLQYs of prepared ICG–BSA NC with different ICG content in 1× PBS (pH 7.4)

Content (wt)	0.1%	0.2%	0.5%	1%	2%	5%
PLQY	0.154	0.155	0.168	0.157	0.143	0.113

The PLQYs were determined by using free ICG in 1× PBS buffer (pH 7.4) as a reference (PLQY = 0.027) [28]

**Fig. 3:**
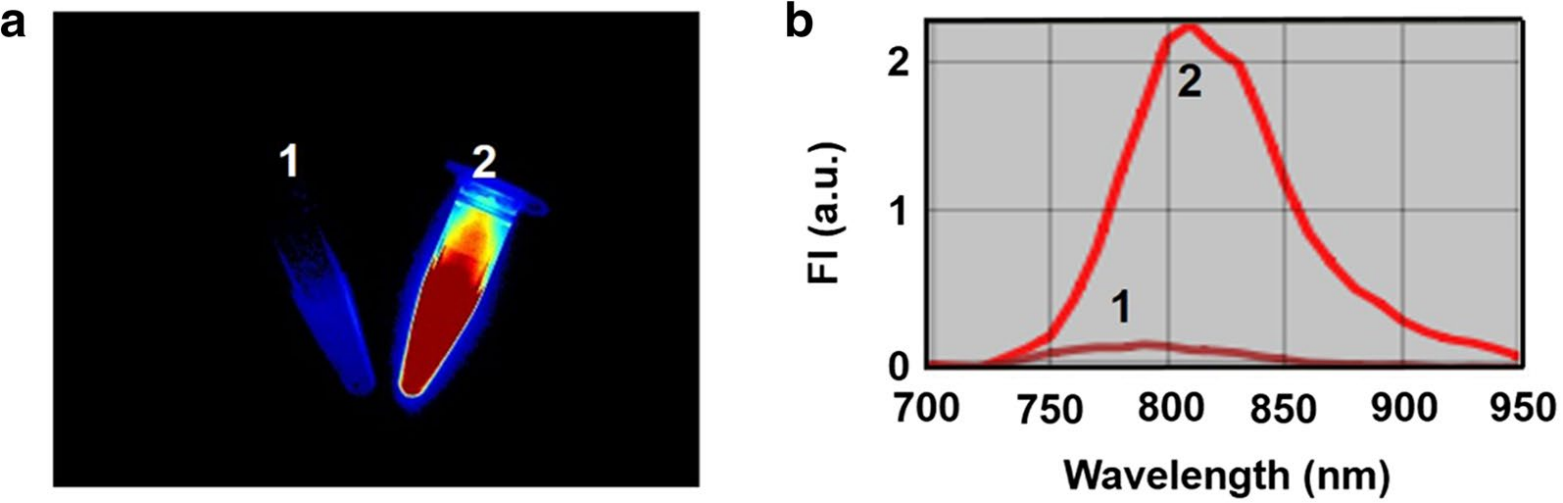
**a** The fluorescence imaging of free ICG and ICG–BSA NC (0.5% ICG content) solution samples (2.8 µg/mL, counted by ICG content) under a 704 nm laser irradiation in a Maestro™ in vivo fluorescence imaging system. **b** The fluorescence spectra of free ICG and ICG–BSA NC solution samples (2.8 µg/mL, counted by ICG content) under a 635 nm laser irradiation in a Maestro™ in vivo fluorescence imaging system. 1—Free ICG solution, 2—ICG–BSA NC solution

In order to confirm the advantage of optimized fluorescence property of ICG–BSA NC, ICG aqueous solution (2.8 µg/mL) and ICG–BSA NC aqueous solution (2.8 µg/mL, counted by ICG content, 0.5% ICG content) were imaged together under a 704 nm laser irradiation in a Maestro™ in vivo fluorescence imaging system. As shown in Fig. 3a, the ICG solution shows weak fluorescence, while ICG–BSA NC solution shows a much stronger fluorescence at the same imaging condition (Fig. 3a). The improved fluorescence emission property is further confirmed with fluorescence spectra that is a function the imaging system equips with. In the fluorescence spectra, the ICG–BSA NC solution exhibits significantly stronger emission profile than that of free ICG solution (Fig. 3b). The fluorescence spectra confirmed the overwhelming advantage of ICG–BSA NC over free ICG for improved NIR FI.

### Hydrolytic stability

**Fig. 4:**
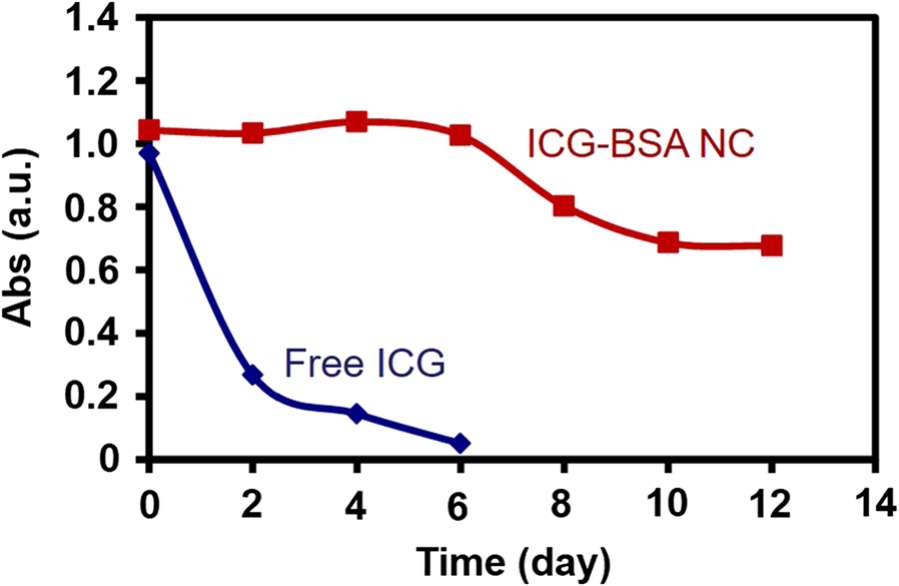
The absorption intensity variations of free ICG and ICG–BSA NC in 1× PBS (pH 7.4). The absorptions intensities are adjusted to ~ 1.0 at the first day and measured every other day

Although ICG is a fluorescent dye widely explored in bioimaging, poor hydrolytic stability is still a limitation for its further applications. In this work, the ICG molecule is incorporated into the BSA-based nanoparticle, which avoids its direct contact with surrounding water to some extent. Inspired by this hypothesis, the hydrolytic stability of the prepared ICG–BSA NC was investigated with UV–Vis–NIR absorption spectra to monitor the variation of its absorption. As shown in Fig. 4, the absorption intensity of free ICG water solution rapidly drops to 25% of its original value after 2 days storage, and further drops to almost zero at the 6th day. In contrast, there is no obvious change for the absorption of ICG–BSA NC water solution. Even after 12 days of storage, the ICG–BSA NC water solution still retains ~ 75% of its original value. The significant improvement of ICG–BSA NC in hydrolytic stability demonstrates the hypothesis that the incorporation of ICG in the BSA-based nanocomplex could reduce the hydrolytic rate of ICG and thus result in a higher quality of probe than free ICG. The improved stability would also benefit the long-term storage in the shelf by avoiding the possible denature induced by the environmental humidity.

### Cytocompatibility evaluation and NIR FI in vitro

**Fig. 5:**
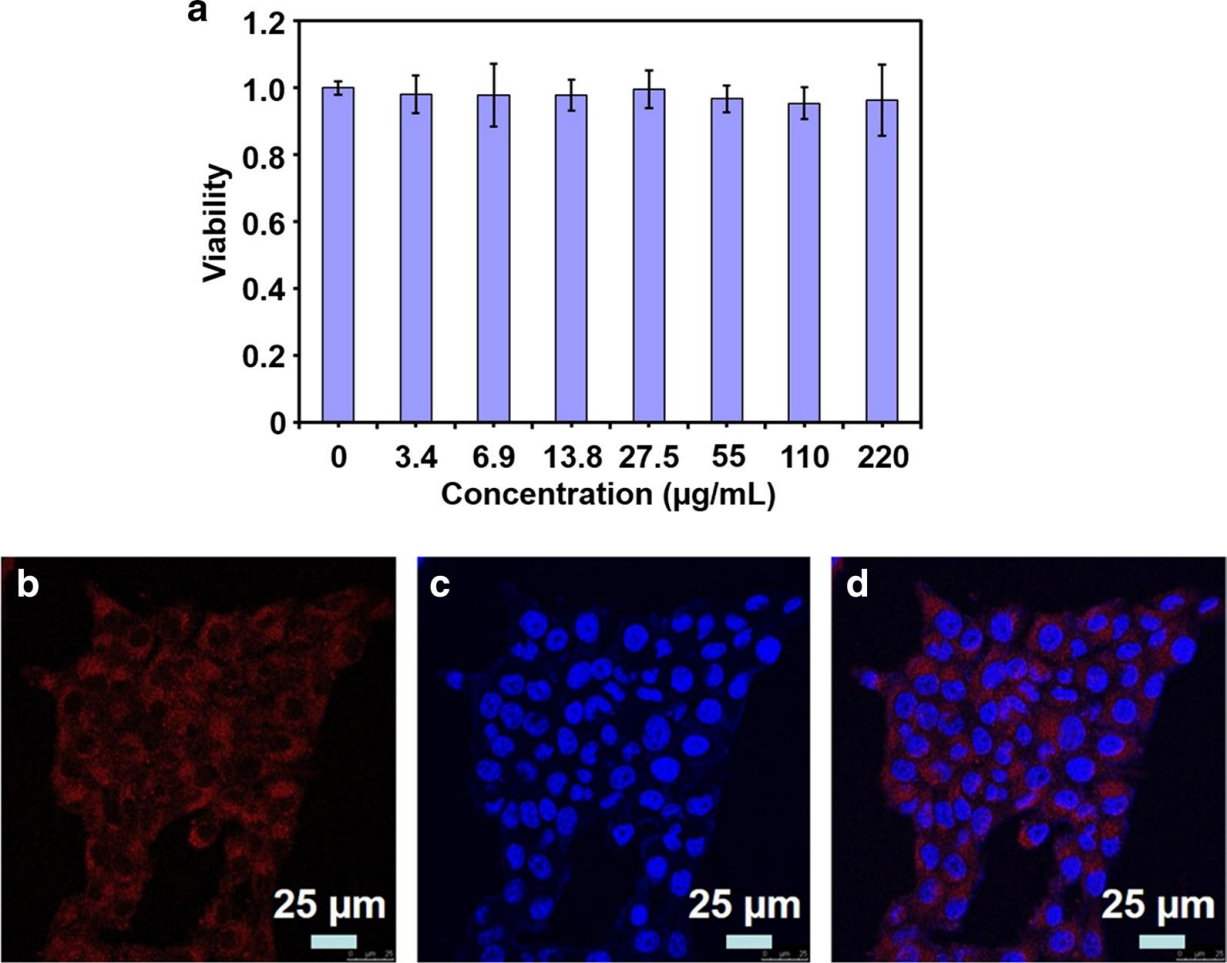
**a** Cytoviability of 4T1 cells after incubation with ICG–BSA NC at different concentrations for 24 h. Data are expressed as mean ± S.D. (n = 3 in each concentration). **b** Image of ICG–BSA NC labeled 4T1 cells captured by CLSM in ICG channel of ICG (Ex/Em = 633/810 nm). **c** Image of DAPI labeled 4T1 cells captured by CLSM in DAPI channel (Ex/Em = 405/480 nm). **d** Overlay of **b** and **c**

To evaluate the cytotoxicity of ICG–BSA NC, triple negative breast cancer 4T1 cells were incubated with ICG–BSA NC at different concentrations for 24 h. The relative viabilities were determined by standard MTT assay. As shown in Fig. 5a, negligible cytotoxicity is observed even at a concentration as high as 220 µg/mL (counted by ICG concentration), indicating that the as-prepared ICG–BSA NC is highly biocompatible and likely safe for FI in vitro and in vivo. The excellent biocompatibility of ICG–BSA NC could be ascribed to the intrinsic biocompatibility of ICG and BSA. The ICG has been in clinic for tens of years with confirmed safety and the albumin is a composition of blood with high biocompatibility. Thus, the ICG–BSA NC that prepared from these two materials inherits their biocompatibility and safety. Furthermore, 4T1 cells were incubated with ICG–BSA NC (3.2 µg/mL, counted by ICG concentration) for 10 h. Then, the cells were stained with DAPI (blue color) for the confocal laser scanning microscope (CLSM) imaging. As shown in the CLSM images, the ICG fluorescence (red color) presents inside 4T1 cells (Figs. 5b–d), indicating the efficient internalization of ICG–BSA NC. The efficient uptake of ICG would contribute to the enhanced fluorescence imaging and efficient PTT on 4T1 cells directly.

### Photothermal conversion and PTT in vitro

**Fig. 6:**
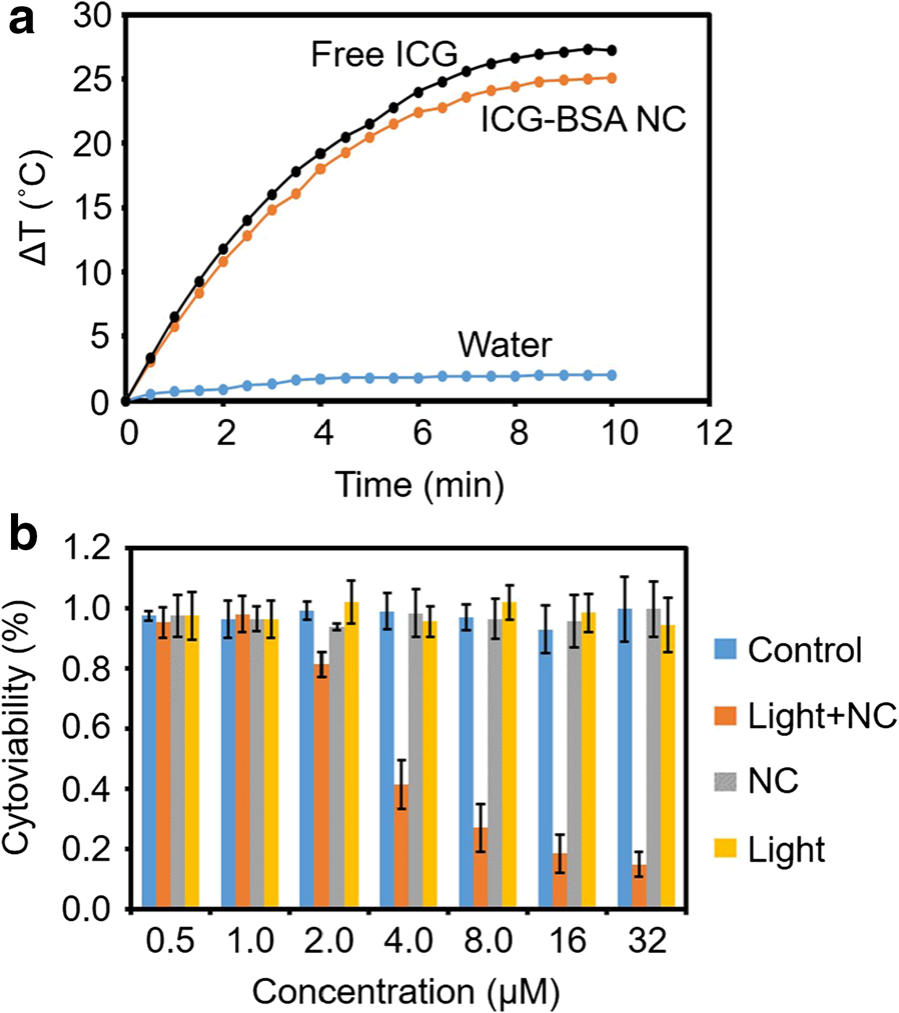
**a** Temperature elevation of water, free ICG and ICG–BSA NC at a concentration of 200 µg/ml (counted by ICG content) under an 808 nm laser irradiation (1 W/cm^2^). **b** The cytoviability evaluation of 4T1 cells under different treatments. In the groups with light irradiation, cells were exposed to an 808 nm laser irradiation for 10 min at 1 W/cm^2^. Data are expressed as mean ± S.D. (n = 3 in each concentration)

Efficient photothermal conversion performance is an important prerequisite to ensure the therapeutic efficacy of a PTT agent. The aqueous ICG–BSA NC solution and aqueous free ICG solution at a concentration of 200 µg/mL were both continuously irradiated with a NIR laser (808 nm, 1 W/cm^2^, 10 min) and the temperature changes were recorded. As shown in Fig. 6a, laser exposure for 10 min resulted in a similar temperature elevation curve for both free ICG and ICG–BSA NC (∆T = 27 °C and 25 °C, respectively), indicating their efficient photothermal conversion efficiency. In contrast, there is only slight temperature increase (~ 2 °C) detected for water that was treated under the same condition. Considering the efficient photothermal effect, ICG–BSA NC could be an effective PTT nanoagent for in vivo tumor therapy.

The in vitro photothermal toxicity of ICG–BSA NC was explored for killing triple negative breast cancer 4T1 cells with or without laser irradiation (808 nm, 1 W/cm^2^, 10 min). The cells were further cultured for another 24 h post laser irradiation and then evaluated with MTT assay for their viability. The MTT assay result shows that ICG–BSA NC treatment without laser irradiation does not induce obvious cell death and the cell viability is more than 95% even at a concentration as high as 32 µg/mL (Fig. 6b). In contrast, the cells treated with both ICG–BSA NC and laser irradiation show an IC50 of ~ 3 µM, indicating that over 50% cells will die under laser irradiation and ICG–BSA NC treatment that has a concentration higher than 3 µM. On the contrary, the cytoviability does not decrease for the cells treated with laser but without ICG–BSA NC treatment. These results indicate that the ICG–BSA NC is an efficient photothermal conversion agent and could be triggered by NIR laser irradiation for efficient in vitro PTT to eliminate cancer cells. The IC50s of all the ICG–BSA NC with different ICG content were also evaluated with MTT assay. The results showed that there is not significant difference in IC50s (~ 3 µM) for all the ICG–BSA NC with different ICG content (Additional file 1: Figure S1). The results demonstrated that the ICG content does not have much impact in the PPT efficacy.

To visually identify the photothermal effect, 4T1 cells incubated with ICG–BSA NC and irradiated laser were stained with Trypan Blue to recognize dead cells (blue). Macroscopic blue spot is outlined in Additional file 1: Figure S2a, preliminarily confirms the laser irradiation induced cell death. The laser irradiated area is observed with microscope and exhibits blue color for all the cell in the region, suggesting efficient cell killing with ICG–BSA NC incubation and laser irradiation (Additional file 1: Figure S2b). At the edge of laser irradiation, only part of cells is stained with blue color and a boundary could be observed (Additional file 1: Figure S2c). In the region without laser irradiation, almost no cell shows blue color, indicating that the cell treated with ICG–BSA NC does not die (Additional file 1: Figure S2d). All the above results demonstrate that the efficient in vitro PTT occurs only under the dual treatments of both ICG–BSA NC and laser irradiation. The results are consistent with the quantitative MTT result and confirm an efficient photothermal nanoagent of ICG–BSA NC for efficient 4T1 cells therapy.

### In vivo NIR FI and biodistribution of ICG–BSA NC

**Fig. 7:**
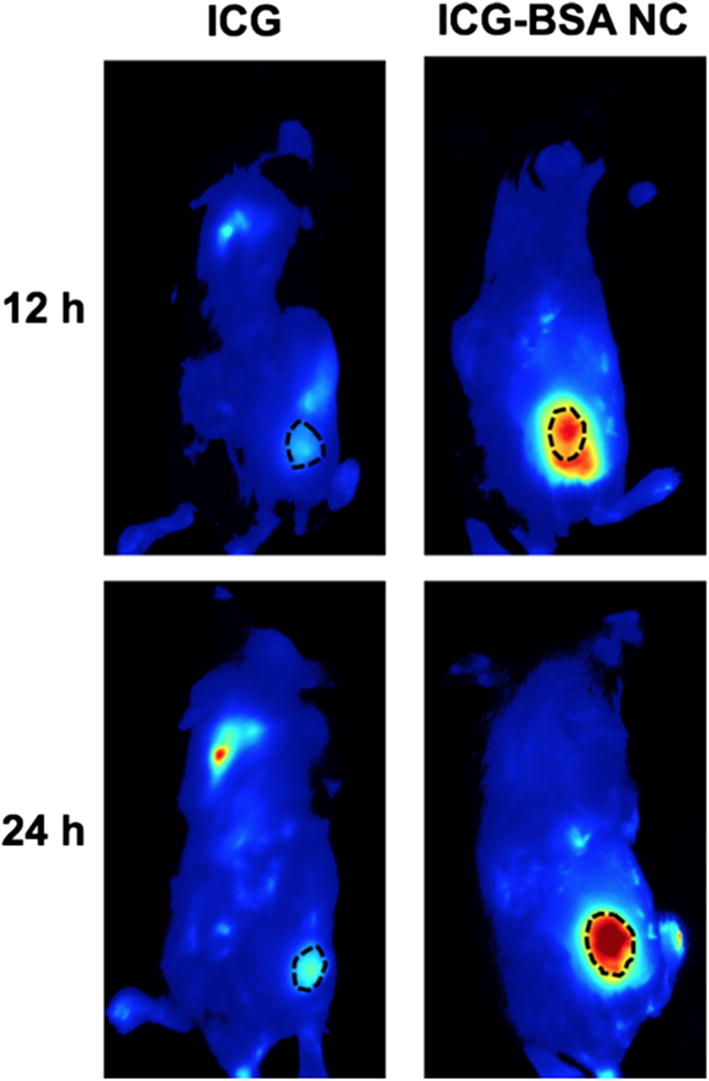
In vivo fluorescence imaging of 4T1 xenograft tumor bearing mice that were *i.v.* injected with ICG and ICG–BSA NC, respectively. The images were captured at 12 h and 24 h post tail vein injection, respectively. The black circles indicate the xenograft tumors

NIR FI was performed to monitor the in vivo biodistribution behavior of free ICG or ICG–BSA NC (10 mg/kg, counted by ICG) in tumors and major organs in the 4T1 tumor-bearing mice. According to the non-invasive NIR FI analysis, the fluorescence signal at tumor site of ICG–BSA NC treated group reaches maximum at 24 h post intravenous injection (Fig. 7b), while the signal in the tumor of free ICG treated group is much weaker (Fig. 7a), indicating an effective tumor accumulation of ICG–BSA NC and a rapid clearance of molecular ICG in vivo. These results demonstrate that the enhanced accumulation of ICG–BSA NC at the tumor site is mainly due to the EPR effect, which is the passive targeting ability for many common nanomaterials with diameter smaller than 200 nm [25]. Furthermore, the diameter of NPs prepared in this study (~ 40 nm) is identified as a suitable size to accumulate and diffuse in dense tumor interstitial matrix, which is supported by the principle that larger NPs with diameter > 60 nm encounter hindered diffusion and smaller NPs with diameter < 20 nm face rapid clearance in vivo [26].

**Fig. 8:**
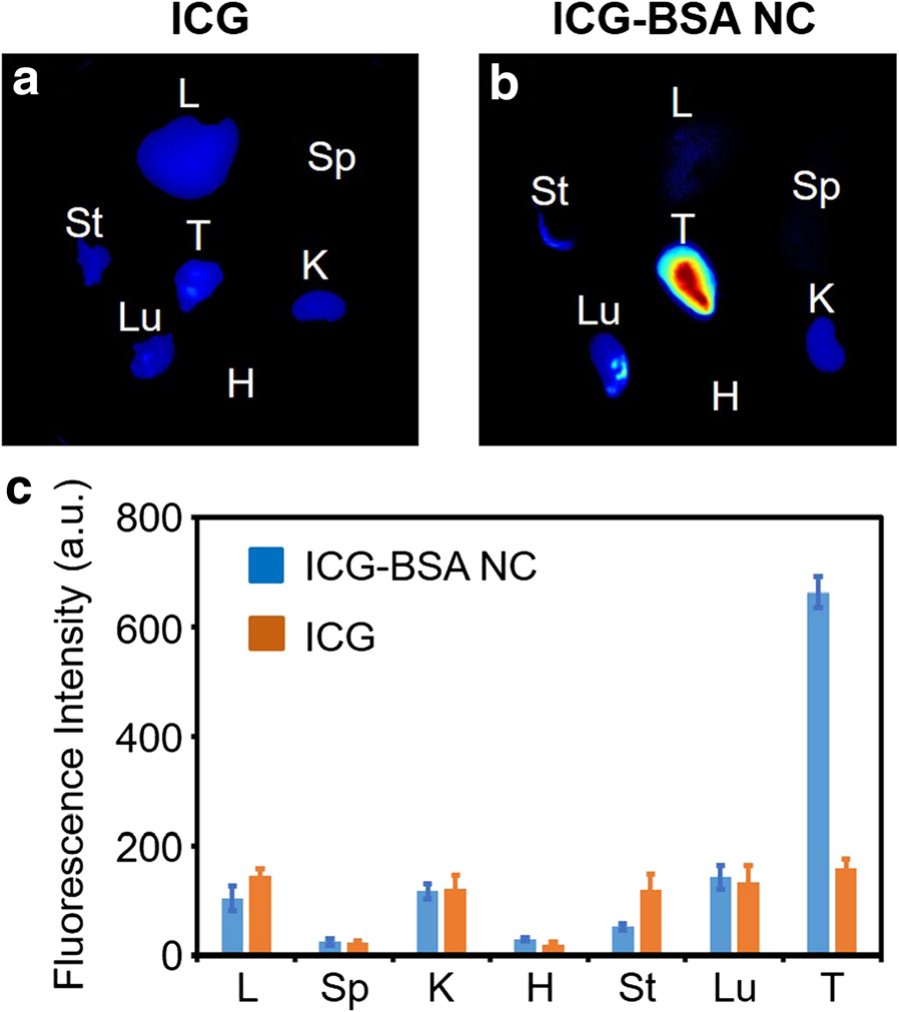
**a** Representative fluorescence image from the ex vivo tumor and organs mapping (24 h post free ICG injection) with Maestro™ in vivo fluorescence system. **b** Representative fluorescence image from the ex vivo tumor and organs mapping (24 h post ICG–BSA NC injection) with Maestro™ in vivo fluorescence system. **c** Biodistribution analysis of free ICG and ICG–BSA NC in mice (3 mice in both groups) by ex vivo organs fluorescence mapping. L—liver, Sp—spleen, K—kidney, H—heart, Lu—lung, St—stomach, T—tumor. Data are expressed as mean ± S.D. (n = 3)

Concentrating nanodrugs at tumor and avoiding accumulation at normal tissues are vital for efficient tumor treatment. Therefore, the in vivo biodistribution of free ICG and ICG–BSA NC were investigated by semi-quantitative analysis with a Maestro™ in vivo fluorescence imaging system. The tumor and major organs were collected and imaged with the fluorescence imaging system 24 h post intravenous injection. The fluorescence signals of all the tissues were extracted and analyzed. As shown in Fig. 8a, weak fluorescence displays on the major organs and tumor in the group treated with free ICG molecule, indicating little distribution of free ICG molecule in the liver, kidney, stomach, lung and tumor. The result is predictable because of the well-known characteristics of ICG for rapid metabolism through liver and kidney. In comparison, the group treated with ICG–BSA NC exhibits strong fluorescence signal at the tumor, which is significantly different from that of free ICG group. The enhanced fluorescence intensity in tumor can be attributed to the EPR effect for passive targeting at the tumor. The efficient tumor accumulation is beneficial for improved in vivo tumor PTT (Fig. 8b). The quantified biodistribution comparison by fluorescence signal statistics of both groups further confirms the efficient accumulation of ICG–BSA NC at the tumor (Fig. 8c), which is highly consistent with the above conclusion. It is well known that liver is a major organ for nanomaterial metabolism and clearance from the body, thus, most of the reported nanomaterials exhibit a higher distribution at the liver than that at the tumor. Interestingly, the liver distribution of ICG–BSA NC is rather low than that at the tumor. The distinctive biodistribution could be ascribed to the reticuloendothelial system-stealthy ability of albumin. The albumin is secreted from the liver that could escape from the liver by itself [27]. The prepared ICG–BSA NC inherits the reticuloendothelial system-stealthy ability of albumin and therefore exhibits little distribution at the liver.

### In vivo antitumor efficacy

**Fig. 9:**
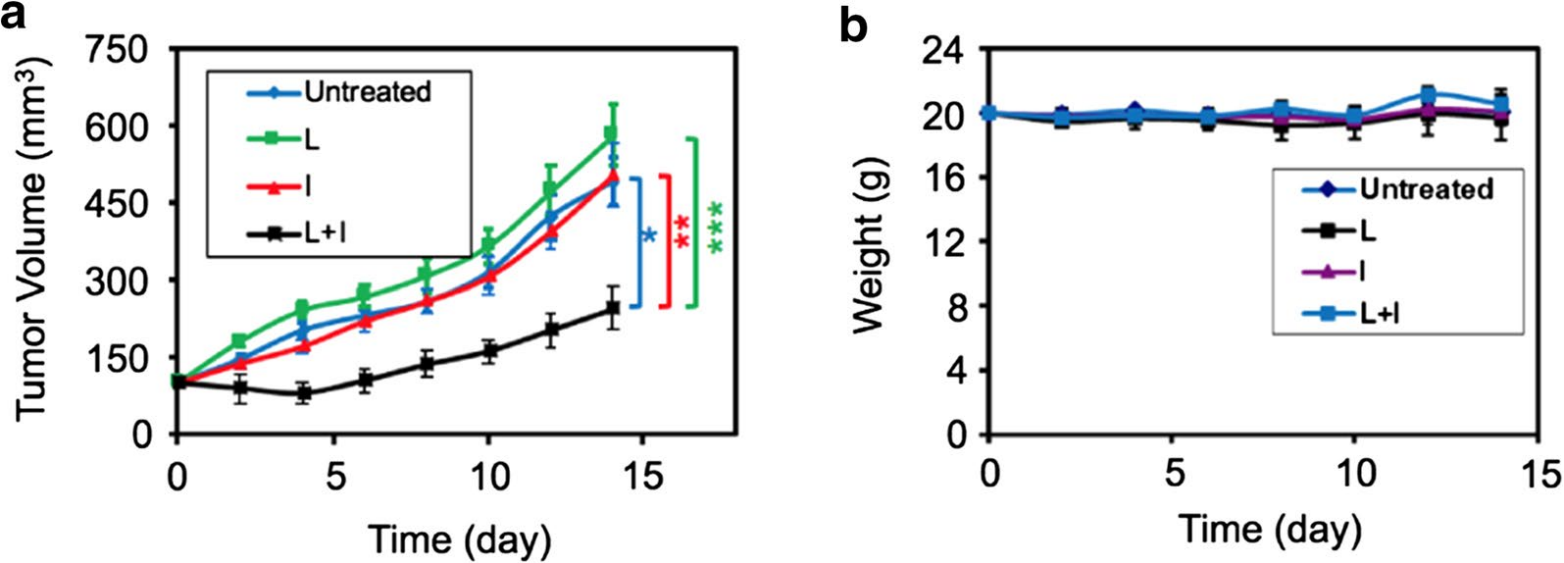
**a** The tumor volume growth curves of mice after various treatments indicated in the 14 days (n = 7 in each group). **b** The body weights of 4T1 tumor-bearing mice after various treatments indicated in the 14 days. Untreated: 1× PBS (pH 7.4); L: Laser irradiation; I: Injected with ICG–BSA NC solution; L + I: Laser irradiation was applied after injecting ICG–BSA NC solution for 24 h. Data are expressed as mean ± S.D. (n = 7) (*P < 0.01, **P < 0.01, ***P < 0.01 by t-test, the tumor growth was significantly inhibited in the group of L + I than that in other groups)

Based on the efficient in vitro PTT and in vivo tumor accumulation results, the ICG–BSA NC is expected to be highly efficient nanoagent for imaging-guided PTT on tumor in living mice. The tumor growth of 4T1 subcutaneous xenograft tumor models was monitored every other day after treatments to determine the antitumor effect of ICG–BSA NC. As shown in Fig. 9a, the tumor volumes of the groups treated with 1× PBS (pH 7.4), laser and ICG–BSA NC increase 5–6 times during the observed 14 days, indicating that the tumor growth was not affected by these treatments. In comparison, the tumor growth is highly inhibited in the group treated with both ICG–BSA NC and laser. Moreover, no severe body weight loss in the mice was observed throughout the therapeutic process, indicating that the treatment is safe and harmless (Fig. 9b). In addition, the skin and major tissues of mice treated with ICG–BSA-laser looked normal compared with that in the PBS group. The representative mice photos were recorded to reflect the tumor change. Owing to the effective accumulation of ICG–BSA NC at the tumor site, the ICG–BSA NC and laser combination treatment induces an apparent tumor ablation and left a black hard scab on the initial tumor location after 3 days’ treatment (Additional file 1: Figure S3). Further evaluation was done by Tunel staining on the tumor slices collected from the indicated groups of mice on day 14. In the Tunel staining images, the group treated with ICG–BSA NC injection and laser irradiation exhibited significantly more green spots, indicator of apoptosis cells, which further demonstrated the efficient PTT treatment with the prepared nanocomplex (Additional file 1: Figure S4). These results confirm that the ICG–BSA NC is a promising candidate for cancer PTT treatment.

### Histology measurement

**Fig. 10:**
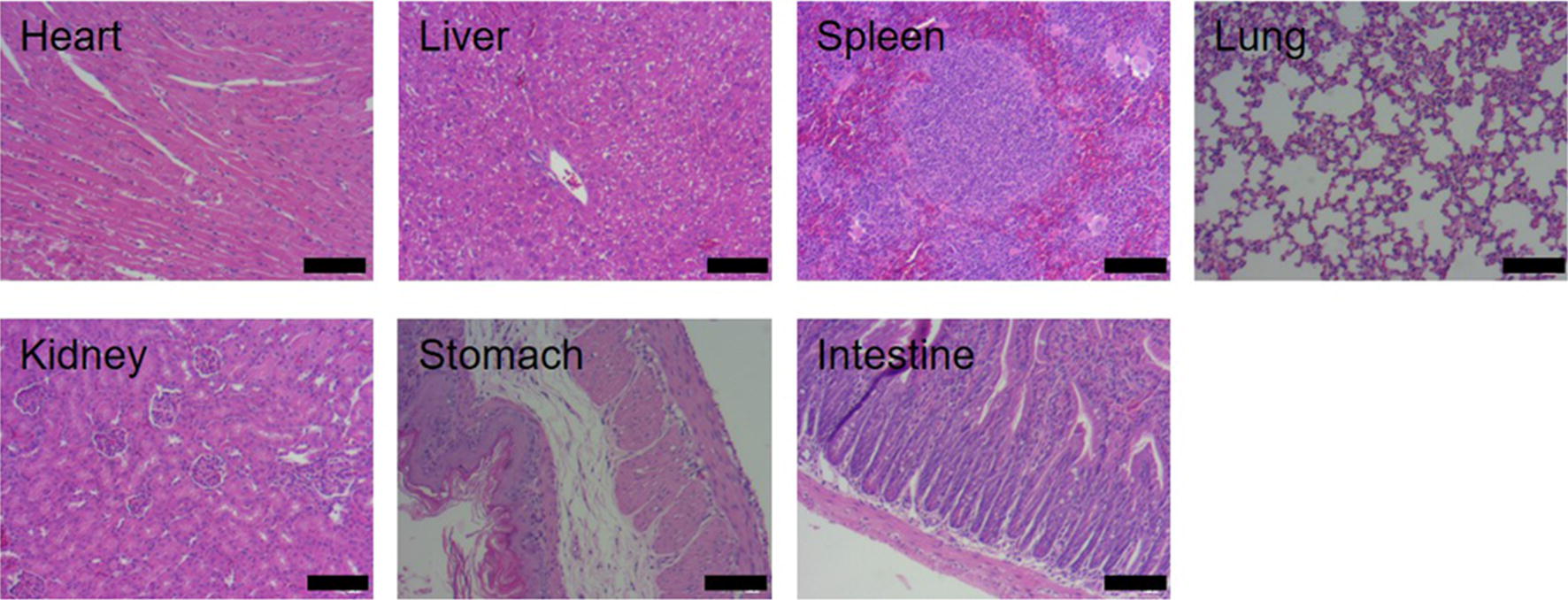
Sections of major organ slices from ICG–BSA NC-treated mice 24 h after intravenous injection with a dose of 10 mg/kg (counted by ICG concentration). Scale Bar = 100 µm

The in vitro cytotoxicity of the ICG–BSA NC against 4T1 cells was evaluated using a standard MTT assay. The results demonstrated that ICG–BSA NC had a negligible influence on the viability of the 4T1 cells up to a concentration of 220 μg/mL (Fig. 5a). The biocompatibility of the ICG–BSA NC in living mice was evaluated through histological examinations on 24 h after intravenous injection with the ICG–BSA NC solution. H&E-stained tissue sections of the heart, liver, spleen, lung, kidneys, stomach and intestine from the ICG–BSA NC-treated groups show no significant organ lesion in morphological observation (Fig. 10).

## Conclusions

In summary, a theranostic nanocomplex that is self-assemble from ICG and BSA is prepared and optimized for enhanced tumor imaging and excellent hydrolytic stability. BSA promoted fluorescence emission of ICG further enhances its fluorescence imaging guided tumor delineation. Benefiting from the EPR effect, intravenously administrated ICG–BSA NC accumulates at the tumor site preferentially via acquired passive targeting and prolonged circulation capability compared with that of free ICG. Furthermore, the imaging-guided cancer PTT indicated an efficient therapeutic outcome, leading to an obvious tumor growth suppression. There is no notable side effects observed in the monitoring of body weight and H&E histology slices of main organs after administrating the NC. The novel ICG–BSA NC would be a promising phototheranostic nanoplatform for highly sensitive NIR FI and imaging-synergized cancer PTT treatment in the future clinical applications. This study reports that the well-proportioned and biocompatible ICG–BSA NPs constitute a new class of tumor-targeted nanomedicine for amplifying the potency of NIR FI guided PTT.

## Materials and methods

### Materials and chemicals

ICG, BSA, ethanol (anhydrous) and dimethyl sulfoxide (DMSO) were purchased from Sigma-Aldrich LLC. 4′,6-diamidino-2-phenylindole (DAPI), RPMI 1640 cell culture medium, 3-(4,5-dimethy ilthiazol-2-yl)-2,5-diphenyltetrazolium bromide (MTT), fetal bovine serum (FBS), trypsin–EDTA solution, penicillin–streptomycin solution and 1× PBS buffer (pH 7.4) were purchased from Sangon Biotech (Shanghai) Co., Ltd. Ultrapure water (18.2 MΩ cm, 25 °C) was used throughout the work.

### Cell culture and animal models

The triple negative breast cancer cell line 4T1 was obtained from American type culture collection (ATCC) and cultured in RPMI 1640 supplemented with 10% (v/v) fetal bovine serum, 1% (v/v) penicillin and 1% (v/v) streptomycin. The cells were incubated in a humidified incubator at 37 °C with 5% CO_2_ atmosphere.

Six-week old female Bal b/c mice (~ 20 g) were purchased from Beijing Vital River Laboratory Animal Technology Co., Ltd. Animals received care in accordance with the Guidance Suggestions for the Care and Use of Laboratory Animals. The procedures were approved by Xi’an Jiaotong University Health Science Center. Xenograft tumor models were established by subcutaneously injecting 100 µL serum free cell culture medium containing 1 × 10^6^ 4T1 cells into the right hind leg of mice. The animal body weight and tumor size were measured every other day. The tumor size was calculated according to the following formula: volume = (length × width^2^)/2. When the volume reached 100 mm^3^, tumor-bearing mice were grouped (n = 7 in each group) and treated with different drug systems.

### Preparation and characterization of ICG–BSA NC

The method was performed according to a modified procedure previously reported reprecipitation method. Briefly, ICG and BSA were dissolved in 20 mL deionized water (DIW) in ratios of 5%, 2%, 1%, 0.5%, 0.2% and 0.1%, respectively. Under vigorous stirring, ethanol (anhydrous) was added dropwise to the mixed solution to form aggregate. The mixture was stirred at 1000 rpm for 1 h in the dark at room temperature and then dialyzed in water (5 KD, 24 h) to remove free ICG and ethanol. Subsequently, the ICG–BSA NPs were collected in the supernatant by centrifugation at 8000 rpm for 10 min to remove the precipitate impurities.

The morphology and structure of the ICG–BSA NPs were characterized by scanning electron microscopy (SEM, FEI Quanta 200F SEM) and transmission electron microscope (TEM, FEI Tecnai G2 F20 S-Twin TEM, Hillsboro, OR). The dynamic diameter and distribution were characterized with dynamic light scattering (DLS) at 25 °C using a Zetasizer Nano-ZS90 (Malvern, UK). The absorption spectra were obtained from a LAMBDA 750 UV/Vis/NIR spectrophotometer (Perkin Elmer). The fluorescence emission spectra were measured with a fluorescence spectrometer (FluoroMax 4, Horiba Jobin–Yvon, Edison, NJ).

### Loading ratio of ICG–BSA NC

The loading ratio of ICG in ICG–BSA NC was calculated as follows: ICG loading (%, w/w) = (ICG weight in ICG–BSA NC/total weight of ICG–BSA NC) × 100%. The loading ratio was optimized for maximal fluorescence intensity. The fluorescence quantum yield (PLQY) was selected as the evaluation index and determined by using free ICG in 1× PBS buffer (pH 7.4) as a reference (PLQY = 0.027) [28]. ICG concentration was determined by UV–vis–NIR absorption spectra. The ICG content corresponding to the maximum PLQY was selected for subsequent physicochemical characterization and theranostic evaluation.

### Hydrolytic stability

The hydrolytic stability of ICG–BSA NC was investigated by measuring the absorption variation of ICG using a LAMBDA 750 UV/Vis/NIR spectrophotometer at scheduled time points. Briefly, the water solution of ICG–BSA NC was diluted and adjusted to absorption intensity of ~ 1.0 at the peak wavelength of ICG and then placed in a transparent quartz cell (light shielding for storage). The absorption intensity of the solution was measured every other day, which continued for 12 days. For comparison, the group of free ICG in DIW was also performed in the same procedure.

### Cytotoxicity assay

A standard 3-(4,5-dimethylthiazol-2-yl)-2,5-diphenyltetrazolium bromide (MTT) assay was employed to evaluate the 4T1 cell viability after 24 h treatment with ICG–BSA NC at different concentrations. 4T1 cells were seeded into 96-well plates with 5 × 10^3^ cells per well (100 µL cell culture medium) and cultured for 24 h (5% CO_2_, 37 °C). Then, fresh RPMI 1640 culture medium that contained ICG–BSA NC at different concentrations (0, 3.4, 6.9, 13.8, 27.5, 55, 110 and 220 µg/mL of final ICG concentrations) was added to replace the old culture medium. After 24 h culture, a standard MTT assay was used to evaluate the relative cytoviability. The cytoviability of the wells that the cells treated with 0 µg/mL ICG–BSA NC was set as 1.0 and the cytoviability of other wells were normalized to it. The experiment was triplicated at each concentration.

### Cellular uptake and NIR FI in vitro

4T1 cells were seeded into cell culture dish (35 mm in diameter) that was equipped with glass (0.13 mm in thickness) at the bottom and incubated for 12 h. Equal amount of culture medium containing ICG–BSA NC (3.2 µg/mL of ICG) was added to culture dish to replace the old culture medium. After 10 h incubation, the medium was discarded and the cells were washed with 1× PBS (pH 7.4) for three times. Then, the cells were stained with DAPI for 10 min, and rinsed three times with 1× PBS (pH 7.4). Cellular uptake and NIR FI were monitored with confocal laser scanning microscope (CLSM, Leica, TCS-SP5) under the DAPI and ICG observation channels that the maximum excitation/emission (Ex/Em) wavelengths were 405/480 nm and 635/810 nm, respectively.

### Photothermal conversion and in vitro PTT

The photothermal effect of ICG–BSA NC was evaluated by detecting its temperature variation (200 μg/mL, counted by ICG concentration) under 808 nm laser irradiation (1 W/cm^2^) for 10 min. The temperature was recorded every 30 s. An equal volume of DIW and free ICG with the same concentration were used as blank and experimental control, respectively.

The in vitro PTT effect of the ICG–BSA NC was first investigated using Trypan Blue staining method. 4T1 cells were seeded in cell culture dish (100 mm in diameter) and incubated for 24 h (5% CO_2_, 37 °C). After removing the old medium, the cells were rinsed with 1× PBS (pH 7.4) and treated with fresh culture medium that contains ICG–BSA NC (8 μg/mL, counted by the concentration of ICG) for 24 h at 37 °C. Subsequently, the cells were irradiated with 808 nm laser aligning the selected area with energy density of 1 W/cm^2^ for 10 min. After another 24 h of culture, the cells were stained with Trypan Blue solution and washed with 1× PBS (pH 7.4) to observe their dead/alive state with naked eyes and a biological inverted microscope.

The photothermal cytotoxicity of ICG–BSA NC was quantified by MTT method. In detail, 5 × 10^3^ 4T1 cells in 100 µL RPMI 1640 cell culture medium were seeded into each scheduled well of 96-well plates and cultured overnight. The cells were divided into four groups as follows: control (without any treatments), treated with ICG–BSA NC, treated with laser only, and treated with both ICG–BSA NC and laser. The ICG concentrations in the groups treated with ICG–BSA NC were 0, 0.5, 1.0, 2.0, 4.0, 8.0, 16.0 or 32.0 µg/mL, respectively. After 24 h, the cells were immersed in 100 μL of fresh medium and underwent laser irradiation or not by 808 nm laser at 1 W/cm^2^ for 10 min. After further 24 h culture, cell viability was measured using MTT assay. The data were presented as the percentage of surviving cells that were calculated according to the absorbance value at 490 nm and reported as the average of three parallel measurements. The experiment was triplicated at each concentration.

### In vivo NIR FI and biodistribution of ICG–BSA NC

For in vivo NIR FI, aqueous solutions of ICG and ICG–BSA NC (10 mg/kg, counted by ICG) were intravenously injected into 4T1 tumor-bearing Bal b/c mice via tail vein when the tumor volume reached ~ 100 mm^3^. The fluorescence signals of ICG were captured by a Maestro™ in vivo fluorescence imaging system (CRI Inc., USA) under 704 nm laser excitation and emission filter that has a longpass of 745 nm (1000 ms exposure and scanned in the wavelength range of 750–950 nm). At 12 and 24 h post-injection, the mice were anesthetized with isoflurane and imaged. At 24 h post-injection, three mice were sacrificed and main organs (heart, liver, spleen, lung, kidney, stomach) and tumor were collected and imaged to evaluate the biodistribution of ICG–BSA NC.

### In vivo PTT

When the tumor size reached ~ 100 mm^3^, 4T1 tumor bearing mice were divided into four groups randomly (n = 7 in each group) as follows: (i) PBS, (ii) laser only (808 nm, 1 W/cm^2^, 10 min), (iii) ICG–BSA NC only (10 mg/kg of ICG) and (iv) ICG–BSA NC + laser (808 nm, 1 W/cm^2^, 10 min). At 24 h post-intravenous injection, the tumor sites were irradiated with a NIR laser (808 nm, 1 W/cm^2^) for 10 min. The laser beam was adjusted to cover the entire tumor region. The tumor volumes were measured every other day during the following 2 weeks. The changes in body weight were also recorded at the same time to evaluate the in vivo side effect. Mice were sacrificed by cervical dislocation under anesthesia after the experiments finished.

### Histology analysis

Three healthy female Bal b/c mice were intravenously injected with ICG–BSA NC solution at a dose of 10 mg/kg (counted by ICG concentration) and sacrificed at 24 h after injection. Major organs from the treated mice were collected for hematoxylin and eosin (H&E) staining according to the standard protocol and examined using an inverted digital microscope (Leica).

## Supplementary information

**Additional file 1: Figure S1.** The MTT assay results of ICG-BSA NC with different loading ratio for PTT on 4T1 cells. **Figure S2.** The photo and microscope pictures of the 4T1 cell seeded culture dish after phototheramal treatment and Trypan Blue staining. **Figure S3.** A representative photo of the 4T1 tumor-bearing mice before and after *i.v.* injected with the ICG-BSA NC and photothermal treatment. **Figure S4.** TUNEL staining images of tumor slices collected from the indicated groups of mice on day 14.

## Data Availability

All data generated or analyzed during this study are included in this published article and its additional file.
