# Supplementary material for: Rationally assembled albumin/indocyanine green nanocomplex for enhanced tumor imaging to guide photothermal therapy
Source: J Nanobiotechnology. 2020 Mar 17;18:49. doi: 10.1186/s12951-020-00603-8 (PMC7079369; doi:10.1186/s12951-020-00603-8)
Supplement: Supplementary file 1 — Additional file 1: Figure S1. The MTT assay results of ICG-BSA NC with different loading ratio for PTT on 4T1 cells. Figure S2. The photo and microscope pictures of the 4T1 cell seeded culture dish after phototheramal treatment and Trypan Blue staining. Figure S3. A representative photo of the 4T1 tumor-bearing mice before and after i.v. injected with the ICG-BSA NC and photothermal treatment. Figure S4. TUNEL staining images of tumor slices collected from the indicated groups of mice on day 14. [file 12951_2020_603_MOESM1_ESM.docx]

**Additional Information**

**Rationally Assembled Albumin/Indocyanine Green Nanocomplex for Enhanced Tumor Imaging to Guide Photothermal Therapy**

Feifei An^1^, Zhao Yang^1^, Meichen Zheng^1^, Ting Mei^1^, Guowei Deng^2^, Ping Guo^1,*^, Yanan Li^3,4,*^, Ruilong Sheng^5^

^1^ Institute of Medical Engineering, Department of Biophysics, School of Basic Medical Science, Health Science Center, Xi’an Jiaotong University, No.76 Yanta West Road, Xi’an 710061, Shaanxi, P. R. China;

^2^ College of Chemistry and Life Science, Institute of Functional Molecules, Chengdu Normal University, Chengdu, 611130, China

^3^ College of Medical Imaging, Shanxi Medical University, Taiyuan 030001, Shanxi, P. R. China;

^4^ Department of Radiology, First Hospital of Shanxi Medical University, Taiyuan 030001, Shanxi, P. R. China;

^5^ CQM-Centro de Quimica da Madeira, Universidade da Madeira, Campus da Penteada, 9000-390, Funchal, Madeira, Portugal

*Corresponding author

Email addresses: guoping6687@xjtu.edu.cn (P. Guo), liyanan0213@163.com (Y. Li)


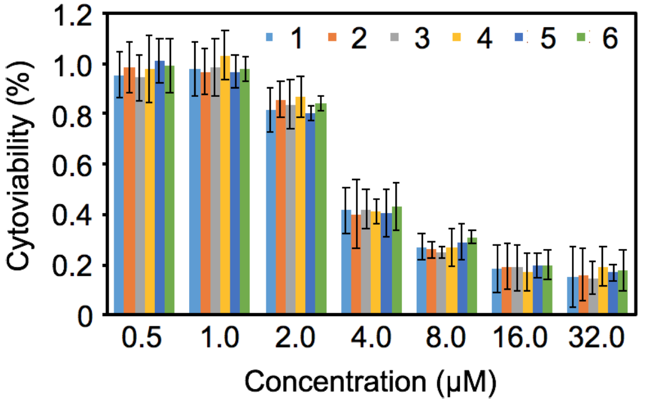


**Figure S1.** The MTT assay results of ICG-BSA NC with different ICG content for PTT on 4T1 cells. 1 - 0.5%; 2 - 0.1%; 3 - 0.2%; 4 - 1%; 5 - 2%; 6 - 5%. The cells in all the groups were irradiated with an 808 nm laser for 10 min at 1 W/cm^2^. Data are expressed as means ± s.d. (n = 3 in each concentration).


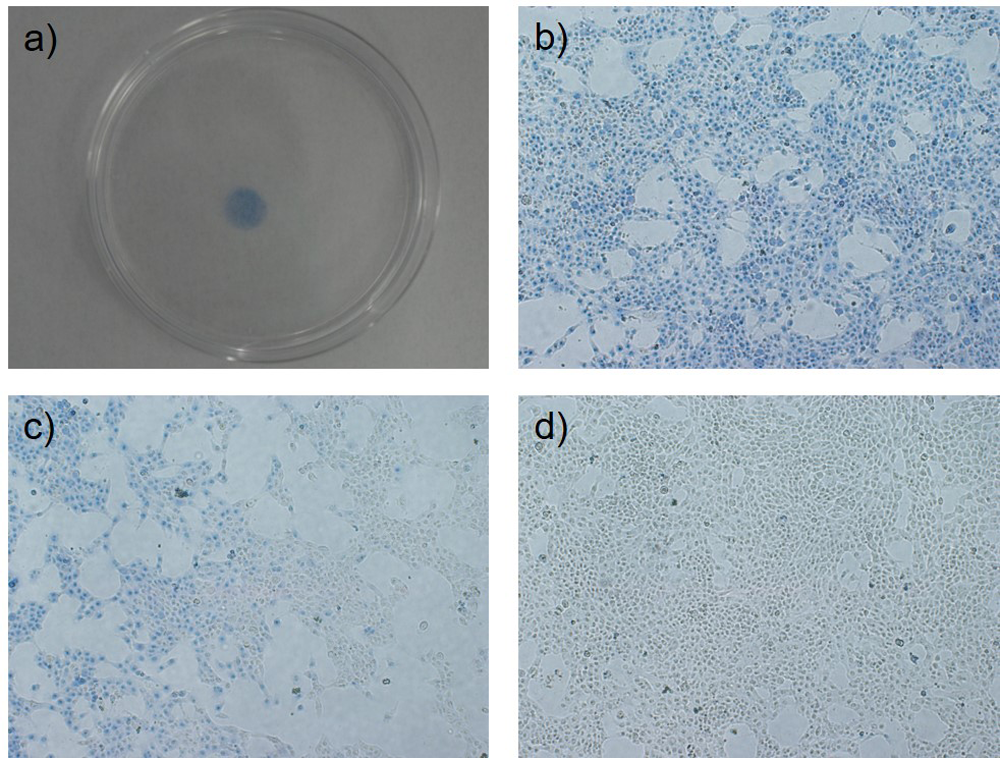


Figure S2. a) The photo of the 4T1 cell seeded culture dish after phototheramal treatment (1 W/cm^2^ for 10 min) and Trypan Blue staining. Blue color is the region with light irradiation and represents dead cells. b) Microscope imaging of the center of the blue region. c) Microscope imaging of edge between the blue region and gray region. d) Microscope imaging of the gray region.


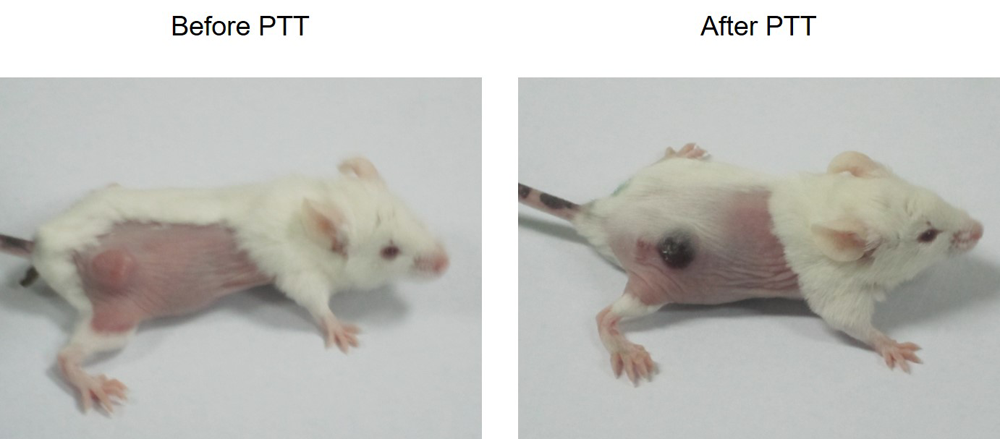


Figure S3. A representative photo of the 4T1 tumor-bearing mice before and after *i.v.* injected with the ICG-BSA NC (1mg/ml, 200 µl) and photothermal treatment (1 W/cm^2^ for 10 min).


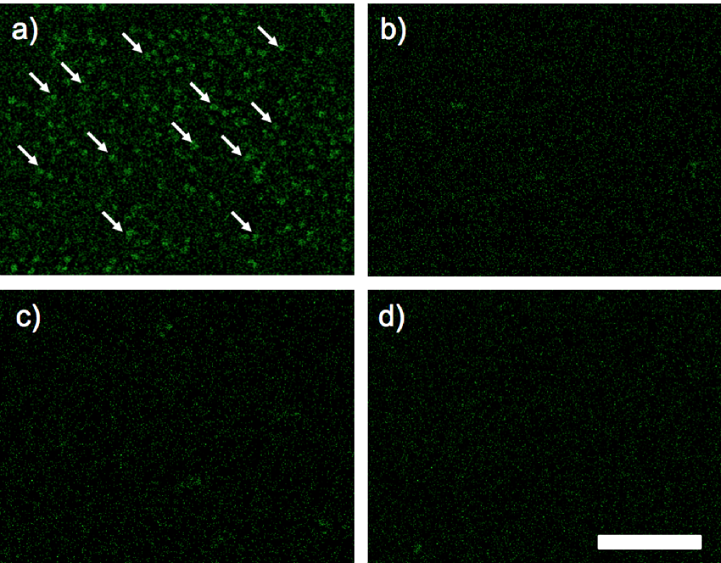


Figure S4. TUNEL staining images of tumor slices collected from the indicated groups of mice on day 14. a) Injection + Laser; b) Injection; c) Laser; d) Untreated. Scale bar = 100 µm. Arrows indicate the apoptosis tumor cells.
